# Daytime napping, biological aging and cognitive function among middle-aged and older Chinese: insights from the China health and retirement longitudinal study

**DOI:** 10.3389/fpubh.2023.1294948

**Published:** 2023-11-17

**Authors:** Huiyi Wu, Lei Huang, Shushan Zhang, Yang Zhang, Yajia Lan

**Affiliations:** ^1^Department of Epidemiology and Biostatistics, West China School of Public Health and West China Fourth Hospital, Sichuan University, Chengdu, China; ^2^West China Hospital/West China School of Medicine, Sichuan University, Chengdu, China; ^3^Department of Neurology, Affiliated Hospital of North Sichuan Medical College, Nanchong, China; ^4^Department of Periodical Press and National Clinical Research Center for Geriatrics, West China Hospital, Sichuan University, Chengdu, China; ^5^Chinese Evidence-Based Medicine Center, West China Hospital, Sichuan University, Chengdu, China; ^6^Department of Environmental Health and Occupational Medicine, West China School of Public Health and West China Fourth Hospital, Sichuan University, Chengdu, China

**Keywords:** cognitive aging, Alzheimer’s disease, neurodegenerative disease, longevity, geroscience, biological aging

## Abstract

**Objective:**

The complicated association of daytime napping, biological aging and cognitive function remains inconclusive. We aimed to evaluate the cross-sectional and longitudinal associations of daytime napping and two aging measures with cognition and to examine whether napping affects cognition through a more advanced state of aging.

**Methods:**

Data was collected from the China Health and Retirement Longitudinal Study. Napping was self-reported. We calculated two published biological aging measures: Klemera and Doubal biological age (KDM-BA) and physiological dysregulation (PD), which derived information from clinical biomarkers. Cognitive z-scores were calculated at each wave. Linear mixed models were used to explore the longitudinal association between napping, two aging measures, and cognitive decline. Mediation analyses were performed to assess the mediating effects of biological age acceleration on the association between napping and cognition.

**Results:**

Participants aged over 45 years were included in the analyses. Non-nappers had greater KDM-BA and PD [LS means (LSM) = 0.255, *p* = 0.007; LSM = 0.085, *p* = 0.011] and faster cognitive decline (LSM = −0.061, *p* = 0.005)compared to moderate nappers (30–90 min/nap). KDM-BA (*β* = −0.007, *p* = 0.018) and PD (*β* = −0.034, *p* < 0.001) showed a negative association with overall cognitive z scores. KDM-BA and PD partially mediated the effect of napping on cognition.

**Conclusion:**

In middle-aged and older Chinese, compared to moderate nappers, non-nappers seem to experience a more advanced state of aging and increased rates of cognitive decline. The aging status possibly mediates the association between napping and cognition. Moderate napping shows promise in promoting healthy aging and reducing the burden of cognitive decline in Chinese middle-aged and older adults.

## 1 Introduction

Aging is commonly characterized as a progressive, generalized impairment in system integrity, manifested as increased vulnerability to disease and death (1). With the projected trends in global population aging, the prevalence of age-related diseases has become a major concern. Currently, dementia has emerged as the greatest challenge in global health and social care (2). In 2019, 55 million people worldwide were estimated to be living with dementia, a number that is projected to rise to 139 million by 2050 (3). Previous research has shown that moderate napping duration (e.g., 30–90 min) is associated with better cognitive performance (4). On the other hand, no naps or overly long naps (e.g., >90 min) may be detrimental (5). However, most of the evidence comes from older adults, especially those aged over 65 years, many of whom are living with chronic illness or experiencing cognitive impairment. Since there is increasing evidence that active interventions for risk factors in midlife may reduce the incidence of dementia, especially Alzheimer’s disease (AD) (2, 6, 7), it is necessary to know whether these associations still exist in the middle-aged and older population, which is essential for early intervention or delaying the dysfunction and AD (8).

As the greatest risk factor, age is strongly associated with an increased risk of dementia, with incidence rates increasing exponentially after the age of 65 (2). No effective medical treatment is currently available for dementia, and as a result, researchers have sought to prevent and intervene in cognitive impairment by understanding its pathophysiological mechanisms and modifiable risk factors. The geroscience hypothesis suggests that interventions to delay the biological processes of aging could prevent age-related diseases and extend healthy lifespan (9, 10). Prior studies found that people who experienced an accelerated aging process tended to have poorer cognitive or memory function (8, 11–13). On the other hand, laboratory studies indicated that sleep disorders can affect the biological aging process through various pathways, such as mitochondrial metabolism, DNA damage, telomeric shortening, and chronic inflammation (14). A study in the United Kingdom Biobank cohort found the acceleration of biological aging would be the consequence of sleep quality (including nighttime sleep duration) (15). A new study has revealed that a higher frequency of napping was causally associated with epigenetic age acceleration based on DNA methylation level (16). Given the potential connections, it seems warranted to evaluate the association of napping duration, BA, with cognitive function, however, the current evidence is limited.

In this study, we used two newly validated composite biological age (BA) measures in the Chinese population, Klemera and Doubal biological age (KDM-BA) and physiological dysregulation (PD). We hypothesized individuals who take no naps or excessive naps exhibit worse cognitive function and greater BA compared to those who take moderate naps. We examined the associations among napping during, BA and cognition in the China Longitudinal Study of Health and Retirement (CHARLS), a national cohort study. Then, we investigated the longitudinal effect of baseline napping and BA on cognitive decline during follow-up. Additionally, we examined the mediating role of BA in the relationship between daytime napping and cognitive function to test whether the acceleration of BA is an underlying pathway linking daytime napping to cognitive function.

## 2 Materials and methods

### 2.1 Study design and participants

CHARLS is a nationally representative longitudinal survey of the middle-aged and older population in mainland China. The baseline survey comprising individuals aged 45 years and older was conducted between June 2011 and March 2012, and three follow-up visits were conducted in 2013 (wave 2), 2015 (wave 3), and 2018 (wave 4). A multistage probability sampling method was used to select interviewees. Data on demographic characteristics, health circumstances, socioeconomic status, and other health-related information was obtained from individual interviews and physical measurements. Whole blood specimens were collected during the baseline and wave 3 surveys. More details on the data have been previously described (17). All participants in the CHARLS study were required to provide signed informed consent. The Ethics Committee of Peking University approved this study (IRB00001052–11015).

In brief, we included participants who provided blood samples at baseline (*N* = 11,847) and restricted to those aged 45–99 years to ensure that individuals were sufficiently old to detect age-related variants in biomarkers but not too old as to represent a chosen population with above-average health (*N* = 1,1,416) (18, 19). Next, we excluded individuals whose information on one or more biomarkers was missing (*N* = 2,205). Participants diagnosed with memory-related diseases and those with missing data on napping, cognition, and other covariates were excluded (*N* = 2,564). Finally, 6,647 respondents were enrolled in the cross-sectional analyses. A total of 6,031 respondents who had at least one remeasurement of cognitive function at follow-up were enrolled in the longitudinal analyses (Supplementary Figure S1).

### 2.2 Exposure

Baseline daytime napping, irrespective of napping frequency, was assessed by asking, “During the past month, how long did you take a nap after lunch?” We obtained information on the duration of one napping session at a certain time of the day (after lunch) for all participants. According to the previous evidence, participants in our study were divided into four groups according to their napping duration at baseline: non-nappers (0 min), short nappers (≤30 min), moderate nappers (30–90 min), and extended nappers (≥90 min). The moderate napping group was set as the reference in our analysis, as moderate nappers showed better cognitive function in previous studies (4, 5, 20).

### 2.3 Biological age measures

We calculated composite BA using two published algorithms: the KDM (18) and PD (21). Based on these two algorithms, KDM-BA and PD have been developed and used in research on senescence and age-related diseases (22, 23). In a recent study, the two BA measures were validated in a Chinese population and demonstrated robust prediction of mortality and disease counts (19). The KDM algorithm is derived from a series of regressions of each individual’s biomarkers on chronological age (CA) in a reference population (24). PD was derived from the Mahalanobis distance by extracting information on multiple biomarkers with respect to a reference or mean baseline population. Unlike KDM-BA, PD does not directly estimate BA and its value represents how anomalous each individual’s physiological profile is compared with a reference sample. PD increases over time within individuals, with higher values indicating an advanced state of aging.

Considering the availability and correlations with CA, a total of eight biomarkers used to estimate KDM-BA and PD for individuals were obtained from blood and physical examinations at the baseline survey and were similar to the biomarker set employed by Liu for the validation analysis in the CHARLS (19). The biomarker list included creatinine, high-sensitivity C-reactive protein, total cholesterol, triglycerides, glycosylated hemoglobin, urea nitrogen, platelets, and systolic blood pressure. The KDM-BA algorithm parameters were trained using data from the China Nutrition and Health Survey (CHNS) cohort and then projected onto CHARLS. For PD, we selected a sample aged 20–39 years from the CHNS as the reference population to parameterize the algorithms, as in previous studies (19, 25). More details on the CHNS have been presented elsewhere (26). The BA measures mentioned above were calculated using the BioAge R package (27). For KDM-BA, we calculated the residuals, referred to as KDM-BA acceleration (KDM-BAacc), resulting from a linear regression model when regressing KDM-BA on the CA for each participant. A value greater than 0 indicates advanced biological age beyond that expected in terms of CA, and values less than 0 are reversed. For PD, the primary statistical distance was log-transformed for approximate normalization and standardized in subsequent analyses.

### 2.4 Outcome

According to previous publications, cognitive function comprising the three aspects of memory, orientation, and executive function was assessed in all CHARLS waves through a battery of tests, including some components of the Telephone Interview of Cognition Status, word recall, and picture drawing (28–31). In brief, the memory function was measured by immediate and delayed word recall for 10 unrelated words, and the memory score was the sum of two individual test scores, ranging from 0 to 20. For the orientation function, respondents were asked to tell investigators the day of the week, date of the month, month of the year, and year. One point was awarded for each correct answer. The executive function test contained two items of the serial sevens test, that is, serial subtraction of 7 from 100 (up to five times, ranging from 0 to 5 points), and a picture drawing test, in which respondents were required to redraw a picture of two intersecting pentagons shown to them, three points were given for a successful drawing and 0 points for a failed drawing. The total scores for orientation and executive function ranged from 0 to 4 and from 0 to 8, respectively. These tests have been demonstrated to be valid and have been widely used in previous studies on cognition in the CHARLS population and others (28–33).

To compare across cognition tests, *z* scores of each domain at each wave standardized to baseline were generated by subtracting the mean score at baseline from each participant’s scores at any wave and dividing by the standard deviation (SD) of the baseline scores. We calculated the global cognitive *z* scores for an individual at each wave by averaging the *z* scores of the three domains and re-standardizing them to the baseline global *z* scores. This approach has been widely adopted (29, 31–34).

### 2.5 Covariates

Potential confounders related to napping, cognition and biological aging were selected for analysis (4, 5, 19, 25, 29, 35). All covariates were collected during the first study visit, including age, sex, residence, education level, body mass index (BMI), marital status, current smoking status, alcohol consumption, night sleep duration, depressive symptoms, and self-reported chronic diseases such as hypertension, diabetes, heart diseases and stroke. Residents were divided into those living in urban and rural areas. Education was divided into three levels: low (elementary school or below), middle (middle school, high school, and vocational school), and high (associate’s degree or above). Weight and height for BMI calculation were measured by objected physical examination. Marital status was classified as married (living with spouse present) or others (including separated, divorced, widowed, and never married). All participants were divided into current smokers and nonsmokers. Alcohol consumption was divided into non-drinkers, drinking less than once a month, and drinking more than once a month. Night sleep duration was assessed using the question, “During the past month, how many hours of actual sleep did you get at night (average hours for one night)?” Participants were divided into three groups: short (<7 h), moderate (7–8 h), and long (>8 h). The ten-item Center for Epidemiologic Studies Depression Scale was used to evaluate depressive symptoms. Each item was scored from 0 to 3, with the sum ranging from 0 to 30, and a score of 12 or higher was defined as depressive symptoms (36). Chronic disease history was measured by self-reporting at baseline.

### 2.6 Statistical analyses

The characteristics of the participants at baseline were described with mean and SD for continuous variables and count and percentages for categorical variables, respectively. The Kruskal–Wallis test or analysis of variance was used for continuous variables, and the chi-square test was used for categorical variables to examine differences across napping duration groups.

We used analyses of covariance to calculate the mean difference across groups to evaluate the association between napping duration and cognitive *z* scores, as well as napping duration and BAs at baseline after adjusting for potential confounders, respectively. The results were expressed as least-square means (LSM) of cognitive *z* scores or the values of two BA measures compared with the reference group (moderate nappers). Multivariable linear model was used to evaluate the association between KDM-BAacc/PD and cognitive *z* scores at the baseline. Model 1 was adjusted for age and sex, and Model 2 was additionally adjusted for the aforementioned covariates.

To further examine the associations of baseline napping and the two BA measures with cognitive decline, we fitted a set of linear mixed models in which the cognitive *z* scores were included as a longitudinal outcome. The models incorporated all available follow-up data and treated missing data as missing at random. A random intercept and slope for each individual were considered to account for the remeasurement of cognitive function and to allow for individual differences at baseline and varying rates of cognitive decline over time. Specifically, the primary models included baseline napping duration, time (years from baseline to each subsequent longitudinal cognitive measure), and the interaction term between napping duration and time. The coefficient of the interaction term manifested the mean difference in the rate of change in cognitive *z* scores (SD/year) compared with the reference group. Model 1 was adjusted for age and sex, and Model 2 was further adjusted for the covariates mentioned above. We hypothesized that greater BA would be associated with faster rates of decline in both global cognitive and three cognitive domains. Additional models (Models 3a and 3b) were further adjusted for two BA measures and the interactions between BA measures and time, based on Model 2, respectively.

Mediation analyses were conducted to assess the effects of BA measures on the association between napping duration and cognitive function. The estimation was performed using the medication R package with bootstrapping (1,000 simulations). The parameter estimates and proportion mediated (the proportion of napping duration effect on cognitive function medicated through BA) were documented.

We also conducted some additional analyses. Firstly, given that the statistically significant association of napping duration with BA was only observed between the non-nappers and the reference group, all respondents were dichotomized into two subgroups, non-nappers and nappers, to assess the effects of napping on cognitive function and BA acceleration, and to set non-nappers as the reference group. Secondly, as the potential non-linear association between daytime napping and cognition, the data were smoothed and then a restricted cubic spline model was used. We selected the model with the smallest AIC to determine the number of knots, and there are 4 knots in the final model. Thirdly, considering that family clustering existed in the CHARLS participants, we performed a sensitivity analysis to further adjust for family effect in our models. Statistical significance was considered to be *p* < 0.05. All statistical analyses were 2-sided and conducted using R version 4.1.1.

## 3 Results

### 3.1 Baseline characteristics and sample size

Among the 6,647 participants included in our study, 3,363 individuals (50.6%) were male. The mean chronological age (SD) of the sample was 58.3 (8.8) years with an average KDM-BA of 57.3 (9.2) years and an average PD of 0.71 (0.38) (Table 1). The Pearson correlation coefficients of KDM-BA and PD with CA were 0.95 and 0.15, respectively (Supplementary Figure S2). The detailed distribution of BA and the correlation of BA with CA in each napping group can be found in Supplementary Figure S3. A total of 6,031 respondents were enrolled in the longitudinal analyses over a median of 7.0 years of follow-up (Supplementary Table S1). The original scores of cognitive domains during follow-up were presented in Supplementary Table S2.

**Table 1:** Baseline characteristics of overall participants and napping duration subgroups.

Characteristics	All	Non-nappers	Short nappers	Moderate nappers	Extended nappers	F/I^2^ value	*p* value
	(*N* = 6,647)	(*N* = 3,017)	(*N* = 1,159)	(*N* = 1707)	(*N* = 764)		
Age	58.3 ± 8.8	57.8 ± 8.5	58.6 ± 8.9	58.6 ± 9.1	59.1 ± 9.3	14.2	0.003
Gender (male)	3,363 (50.6)	1,337 (44.3)	586 (50.6)	996 (58.3)	444 (58.1)	105.9	<0.001
Residence (urban)	2,489 (37.4)	1,065 (35.2)	473 (40.8)	664 (38.9)	287 (37.6)	13.1	0.004
Education level^a^							
Low	4,327 (65.1)	2062 (68.3)	698 (60.2)	1,075 (63.0)	492 (64.4)	33.8	<0.001
Middle	1,681 (25.3)	699 (23.2)	335 (28.9)	441 (25.8)	206 (27.0)		
High	639 (9.6)	256 (8.5)	126 (10.9)	191 (11.2)	66 (8.6)		
Marital status (married)	5,675 (85.4)	2,579 (85.5)	976 (84.2)	1,484 (86.9)	636 (83.2)	7.4	0.060
BMI (kg/m^2^)^b^							
<18.5	378 (5.7)	194 (6.4)	53 (4.6)	97 (5.7)	34 (4.5)	37.3	<0.001
18.5–23.9	3,446 (51.8)	1,650 (54.7)	573 (49.4)	847 (49.6)	376 (49.2)		
24–28	2017 (30.3)	850 (28.2)	365 (31.5)	554 (32.5)	248 (32.5)		
≥28	806 (12.1)	323 (10.7)	168 (14.5)	209 (12.2)	106 (13.9)		
Current smoking	2,811 (42.3)	1,168 (38.7)	463 (39.9)	804 (47.1)	376 (49.2)	49.6	<0.001
Drinking status							
Non-drinker	4,321 (65.0)	2086 (69.1)	756 (65.2)	1,024 (60.0)	455 (59.6)	59.3	<0.001
Less than once a month	543 (8.2)	207 (6.9)	112 (9.7)	163 (9.5)	61 (8.0)		
More than once a month	1783 (26.8)	724 (24.0)	291 (25.1)	520 (30.5)	248 (32.5)		
Night sleep duration							
<7 h	3,335 (50.2)	1,618 (53.6)	601 (51.9)	794 (46.5)	322 (46.9)	52.9	<0.001
7-8 h	2,802 (42.2)	1,176 (39.0)	482 (41.6)	786 (46.0)	358 (42.1)		
>8 h	510 (7.7)	223 (7.4)	76 (6.6)	127 (7.4)	84 (11.0)		
Depressive symptoms^c^	1740 (26.2)	886 (29.3)	283 (24.4)	398 (23.3)	173 (22.6)	29.9	<0.001
Chronic diseases							
Hypertension	1,657 (25.0)	636 (21.1)	342 (29.5)	471 (27.6)	208 (27.2)	45.5	<0.001
Diabetes	398 (6.0)	140 (4.6)	92 (7.9)	120 (7.0)	46 (6.0)	20.9	<0.001
Heart disease	776 (11.7)	303 (10.0)	180 (15.5)	213 (12.5)	80 (10.5)	26.6	<0.001
Stoke	129 (1.9)	50 (1.7)	25 (2.2)	33 (1.9)	21 (2.7)	4.15	0.245
Memory score	7.4 ± 3.3	7.2 ± 3.3	7.7 ± 3.3	7.5 ± 3.3	7.1 ± 3.2	8.1	<0.001
Orientation score	3.1 ± 1.0	3.1 ± 1.0	3.2 ± 1.0	3.2 ± 0.9	3.1 ± 1.0	34.3	<0.001
Executive score	5.8 ± 2.4	5.6 ± 2.5	5.9 ± 2.3	6.0 ± 2.3	5.8 ± 2.3	38.3	<0.001
KDM-BA	57.3 ± 9.2	56.8 ± 8.8	57.4 ± 9.3	57.6 ± 9.5	58.2 ± 9.5	14.5	0.002
PD	0.71 ± 0.38	0.73 ± 0.37	0.71 ± 0.38	0.68 ± 0.38	0.71 ± 0.38	8.4	<0.001

BMI, body mass index; ADL, activities of daily living; IADL, instrumental activities of daily living; KDM-BA, Klemera and Doubal method-biological age; PD, physiological dysregulation.

Data are expressed as mean ± standard deviation (SD) for continuous variables and as count(percentage) for categorical variables.

^a^Low education level was defined as elementary school or below; middle was defined as middle school, high school, and vocational school; and high was defined as an associate degree or above.

^b^BMI was calculated as weight in kilograms divided by height in meters squared.

^c^Depressive symptoms were defined as a ten-item Center for Epidemiologic Studies Depression (CES-D 10) scale score of 12 or above.

### 3.2 Cross-sectional associations of baseline daytime napping and biological age measures with cognitive function

After adjusting for possible confounders, the LSM of the global cognitive *z* scores were lower in the non-napper and extended napper subgroups than in the moderate napper subgroup (Table 2). These associations were also observed in orientation function but not memory function and executive function (Supplementary Table S3). The associations of two BA measures with global cognition and *z* scores of the three domains are presented in Table 3. After adjusting for age and sex (Model 1), the baseline KDM-BAacc was negatively associated with global cognitive and memory *z* scores (*β* = −0.008, 95% CI: −0.014, −0.002; *β* = −0.010, 95% CI: −0.018,-0.002), and PD was negatively associated with global cognition and all specific domains. These associations persisted after controlling for more covariates, except for the association of PD with memory function (Model 2).

**Table 2:** Cross-sectional associations of baseline napping duration with global cognitive function or biological age measures.

Napping group	Global *z* scores		KDM-BAacc		PD	
	LSM^a^ (95%CI)	*p* value	LSM^a^ (95%CI)	*p* value	LSM^a^ (95%CI)	*p* value
Non-nappers (0 min)	-0.061 (−0.106, −0.015)	0.005	0.255 (0.058,0.451)	0.007	0.085 (0.016,0.154)	0.011
Short nappers (≤ 30 min)	0.012 (−0.045,0.068)	0.899	−0.179 (−0.424,0.064)	0.202	0.031 (−0.056,0.115)	0.701
Moderate nappers (30–90 min)	Reference	–	Reference	–	Reference	–
Extended nappers (≥ 90 min)	−0.078 (−0.143, −0.011)	0.013	0.053 (−0.226,0.333)	0.916	0.067 (−0.031,0.165)	0.257

LSM, least-squares means; KDM-BAacc, Klemera and Doubal method-biological age acceleration; PD, physiological dysregulation; CI, confidence interval.

^a^After adjusting for age, sex, education level, residence, BMI, marital status, current smoking and drinking status, night sleep duration, depressive symptoms, and self-reported chronic diseases.

**Table 3:** Cross-sectional associations of biological age measures with cognitive function.

Cognitive function (*z* scores)	KDM-BAacc				PD			
	Model 1		Model 2		Model 1		Model 2	
	*β* (95%CI)	*p* value	*β* (95%CI)	*p* value	*β* (95%CI)	*p* value	*β* (95%CI)	*p* value
Global	−0.008 (−0.014, −0.002)	0.008	−0.007 (−0.012, −0.001)	0.018	−0.045 (−0.062, −0.028)	<0.001	−0.034 (−0.050, −0.018)	<0.001
Memory	−0.010 (−0.018, −0.002)	0.013	−0.008 (−0.017, −0.000)	0.043	−0.034 (−0.058, −0.010)	0.005	−0.021 (−0.045,0.001)	0.065
Orientation	−0.002 (−0.011,0.005)	0.480	−0.007 (−0.015,0.001)	0.112	−0.050 (−0.074, −0.026)	<0.001	−0.042 (−0.065, −0.018)	<0.001
Executive	−0.007 (−0.015,0.000)	0.067	−0.007 (−0.015,0.001)	0.102	−0.052 (−0.075, −0.028)	<0.001	−0.041 (−0.063, −0.018)	<0.001

KDM-BAacc, Klemera and Doubal method, biological age acceleration; PD, physiological dysregulation; CI, confidence interval. Model 1 was adjusted for age and sex. Model 2 was additionally adjusted for education level, residence, BMI, marital status, current smoking and drinking status, night sleep and napping duration, depressive symptoms, and self-reported chronic diseases.

### 3.3 Longitudinal associations of baseline daytime napping and biological age with cognitive decline

The cross-sectional analysis only assessed the status of cognition as measured at baseline. We further investigated how the duration of napping and biological age at baseline relate to cognition decline during the follow-up. The mean difference in the global cognitive *z* scores and the mean difference in the rate of change in global cognition are presented in Table 4. Non-nappers and extended nappers had an increased decline rate in global cognitive *z* scores compared with moderate nappers, and the multivariable-adjusted rates by −0.013 SD/year (95% CI: −0.024, −0.003) and − 0.020 SD/year (95% CI: −0.036, −0.005), respectively (Table 4). However, no difference in the rate of decline in *z* scores of the three cognitive domains was observed between the napping duration groups (Supplementary Tables S4–S6). We further adjusted for two BA measures and the interaction term of BAs with time in our models (Models 3a and 3b), indicating that higher PD was associated with baseline global cognitive *z* scores and the cognitive decline rate at follow-up (*β* = −0.011,*p* < 0.001) (Table 4).

**Table 4:** Longitudinal associations of baseline napping duration and biological age measures with global cognitive decline.

Variable	Model 1		Model 2		Model 3a		Model 3b	
	*β* (95%CI)	*p* value	*β* (95%CI)	*p* value	*β* (95%CI)	*p* value	*β* (95%CI)	*p* value
Napping duration, min								
0	−0.108 (−0.152, −0.063)	<0.001	−0.051 (−0.095, −0.014)	0.009	−0.053 (−0.093, −0.012)	0.011	−0.051 (−0.092, −0.010)	0.013
≤ 30	0.016 (−0.040,0.072)	0.566	−0.009 (−0.060,0.041)	0.700	−0.011 (−0.061,0.040)	0.675	−0.009 (−0.059,0.042)	0.734
30–90	Reference	–	Reference	–	Reference	–	Reference	–
≥ 90	−0.084 (−0.147, −0.019)	0.011	−0.081 (−0.140, −0.027)	0.007	−0.080 (−0.141, −0.026)	0.008	−0.080 (−0.140, −0.025)	0.005
Time since baseline (years)	−0.060 (−0.068, −0.052)	<0.001	−0.058 (−0.066, −0.050)	<0.001	−0.058 (−0.066, −0.050)	<0.001	−0.059 (−0.068, −0.051)	<0.001
Napping duration x Time since baseline								
0	−0.014 (−0.024, −0.004)	0.008	−0.013 (−0.024, −0.003)	0.012	−0.013 (−0.024, −0.003)	0.012	−0.013 (−0.023, −0.002)	0.016
≤ 30	−0.001 (−0.014, 0.012)	0.936	0.000 (−0.013, 0.013)	0.990	0.000 (−0.013, 0.013)	0.989	0.001 (−0.012, 0.014)	0.923
30–90	Reference	–	Reference	–	Reference	–	Reference	–
≥ 90	−0.021 (−0.036, −0.006)	0.006	−0.020 (0.034, −0.005)	0.007	−0.020 (−0.035, −0.005)	0.008	−0.019 (−0.034, −0.004)	0.012
KDM-BAacc	–	–	–	–	−0.007 (−0.013, −0.001)	0.024	–	–
KDM-BAacc x Time since baseline	–	–	–	–	−0.001 (−0.003,0.000)	0.082	–	–
PD	–	–	–	–	–	–	−0.027 (−0.044, −0.010)	0.002
PD x Time since baseline	–	–	–	–	–	–	−0.011 (−0.016, −0.007)	<0.001

KDM-BAacc, Klemera and Doubal method, biological age acceleration; PD, physiological dysregulation; CI, confidence interval. Model 1 was adjusted for age and sex. Model 2 was additionally adjusted for education level, residence, BMI, marital status, current smoking and drinking status, night sleep duration, depressive symptoms, and self-reported chronic diseases based on Model 1. Model 3a was additionally adjusted for KDM-BAacc, based on Model 2. Model 3b was additionally adjusted for PD, based on Model 2.

### 3.4 The mediating effect of biological age measures

**Figure 1:**
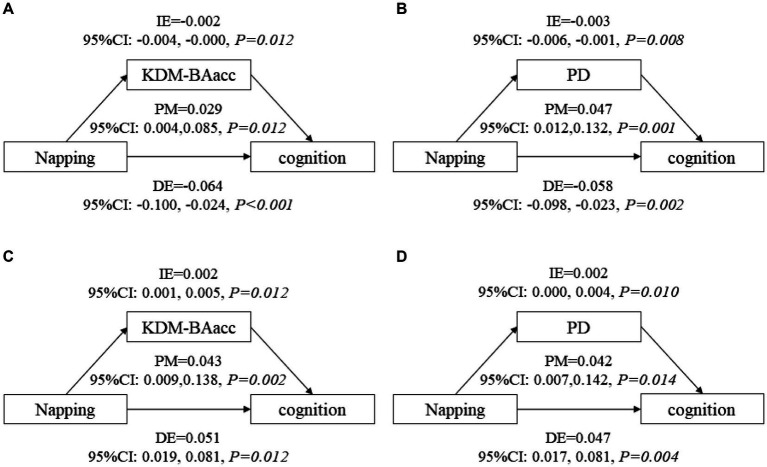
The mediating role of biological age in the association between napping and global cognitive function. KDM-BAacc, Klemera and Doubal method-biological age acceleration; PD, physiological dysregulation; CI, confidence interval; DE, direct effect; IE, indirect effect; PM, proportion-mediated. **(A,B)** show the mediating effects of biological age in main analyses, **(A)** for KDM-BA and **(B)** for PD. **(C,D)** show the mediating effects of biological age in additional analysis, **(C)** for KDM-BA and **(D)** for PD. Model after adjusting for age, sex, education level, residence, BMI, marital status, current smoking and drinking status, night sleep duration, depressive symptoms, and self-reported chronic diseases.

After adjusting for covariates, non-nappers tended to have higher LSM for both BA measures, indicating an advanced biological senescence state compared with moderate nappers. However, the association was not significant for the short and extended nappers (Table 2). Based on the results, non-nappers, but not extended nappers, were associated with greater BA compared with moderate nappers. Hence, we set the non-nappers as the treatment group and the moderate nappers as the control group for comparison in mediation analyses. Considering both greater BA measures were associated with cognitive function at baseline, though it was the PD, not the KDM-BAacc, that predicted cognitive decline during follow-up. This remained relevant for examining the effect of current KDM-BAacc on the association between napping and cognition. Mediation models were performed separately for the two BA measures. KDM-BAacc and PD mediated 2.9 and 4.7% of the napping duration effect on global cognition, respectively, compared to those in the non-napper subgroup and those who reported taking moderate naps (Figures 1A,B).

### 3.5 Additional analysis

All respondents were dichotomized into two subgroups, non-nappers and nappers, cross-sectional and longitudinal analyses suggested that nappers were associated with higher global cognitive *z* scores at baseline (LSM =0.056, 95% CI: 0.022, 0.083) and a decreased rate of change in global cognitive decline (*β* = 0.009, 95% CI: 0.001, 0.017), compared with those who did not take naps (Supplementary Tables S7, S8). Furthermore, non-nappers were associated with higher LSM for both BA measures, similar to the main analysis (Supplementary Table S5). Similarly, mediation models were constructed, and 4.3 and 4.2% of the napping effect on global cognitive function was mediated by KDM-BAacc and PD, respectively (Figures 1C,D). In addition, we exploratory analyzed the nonlinear relationship between daytime napping and cognitive function scores, with the highest scores occurring at 60-70-min naps (Supplementary Figure S4). The effect of family clustering was further examined in sensitivity analysis, which did not alter the significance of the results, confirming the robustness of our findings (Supplementary Table S9).

## 4 Discussion

In this community-based study, we observed that non-napping and excessive daytime napping duration (≥90 min) were associated with a lower cognitive performance level and faster cognitive decline among middle-aged and older Chinese. More importantly, we found that non-napping may be associated with BA acceleration and that acceleration is associated with cognitive function. Our findings suggest that two BA measures, the KDM-BAacc and PD, partially mediate the association between daytime napping and cognitive function.

The results of the cross-sectional and longitudinal analyses on the association of daytime napping with cognitive function were generally consistent, similar to the results of previous studies (4, 37–39). Our results revealed that no napping and excessive napping duration (≥90 min) were associated with worse cognitive function and increased rate of cognitive decline than moderate napping (30–90 min). Interestingly, irrespective of the napping duration, the overall cognitive function of nappers was better than that of non-nappers, and the parameter estimates were much larger than those grouped by napping duration in our main analyses. Sleep is essential for memory consolidation and normal brain function (40). Sleep disorders or sleep deprivation are linked to β-amyloid (Aβ) deposition, increased Tau, and interference with the function of neuronal pathways, especially those of GABA and cAMP, leading to AD (40, 41). Sleep is considered a crucial restorative process for the brain and body, contributing to the recovery of energy and attention, as well as cellular restoration (14).Napping may help compensate for nighttime sleep loss, compared to non-nappers, moderate napping may provide extra resistance to cognitive decline in people who sleep poorly at night (38). On the other hand, excessive daytime sleepiness may be detrimental to maintaining cognitive function. Nevertheless, it is worth noting that the excessive duration cutoff value varied in previous studies, which may have contributed to the inconsistent results (4, 37–39).

As far as we know, our study is the first to evaluate the association between daytime napping, biological aging, and cognitive function in a large prospective cohort of community-dwelling individuals. Our results demonstrated that non-napping was significantly associated with two BA measures established by drawing information on clinical biomarkers from multiple systems in the human body, indicating that non-napping could be a potential risk factor for BA acceleration. A study reported that abnormal (too short or excessive) sleep is implicated in bigger BA, and normal night sleep duration (7-8 h) was associated with 0.245 years decreases in KDM-BAacc (15). Although a new study reported the evidence for causal effects of daytime napping frequency on epigenetic age, and a higher frequency of daytime napping was associated with epigenetic age acceleration (16), more evidence is required on the effects of other napping habits such as duration and timing. A significant association was observed between non-napping and increased biological age in our analyses, it seemingly shed light on the possibility of napping in the resistance to accelerated aging. However, due to the limited evidence, we believe that the results should be interpreted cautiously and that more objective studies are warranted to replicate our results.

People with higher values of KDM-BAacc or PD are more likely to experience worsening cognitive performance. These findings are consistent with those of previous studies (8, 10, 11). Biologically older individuals had poorer cognitive performance at midlife, and this difference mirrored a real decrease in cognitive function over time (8). Notably, our results suggested that the two measures revealed inconsistent associations with specific domains. We observed that PD was negatively correlated with all cognitive domains, whereas KDM-BA was associated only with memory function. PD appeared to be more predictive and sensitive to the unique aspects of cognitive function. One possible reason is that KDM-BA and PD were calculated by two different algorithms that could capture disparate characteristics of biomarkers and quantify the aging process differently (10, 19), although we selected the same set of biomarkers in our calculations.

This study provides initial evidence for using two accepted measures in quantifying biological age as an underlying pathway linking daytime napping to global cognitive function. Sleep disturbances and sleep deprivation could play a direct role in aging mechanisms (14). Current biological mechanisms linking sleep to biological aging, such as inflammation, mitochondrial dysregulation and epigenetic changes, were also thought to be the underlying aging process for cognitive decline and neurological diseases, including ADRD (14, 42). Our results suggest that if we could intervene in changeable risk factors that contribute to an advanced state of biological aging, we might be able to improve cognitive decline even in the preclinical stage of AD. Furthermore, our results highlight the critical role of moderate daytime napping in slowing down the rate of cognitive decline and biological age acceleration, although the magnitude of the effect we observed was limited. The mediation effect sizes tended to be small and insignificant in the three cognitive domains of our study; however, we believe that this effect should not be completely ignored. Previous studies have suggested that inflammatory factors mediate the relationship between sleep-related behaviors and cognitive function (35); however, KDM-BA and PD, have captured clinical profiles of multiple organs and systems, not just inflammation systems. This is promising to understand further the pathways involved in the daytime napping effect on cognition, and our results provide statistical epidemiological evidence for subsequent mechanistic studies.

A strength of our study was the large population size of the Chinese community-based population, which allowed us to obtain sufficient blood data for biological age estimation. In addition, we used parallel analyses of two conceptually diverse composite biological age measures that had been previously trained and validated in the same sample (19), which enhanced the robustness of our conclusions. However, our study also had some limitations. Firstly, the measure of cognition used in this study is rudimentary when compared to a complete neuropsychological evaluation, although the abbreviated nature of the study’s cognitive evaluation made it practical to assess such a large sample size. Secondly, this study is only limited to the investigations of naps taken after lunch. Other dimensions like frequency and complete range of nap timing throughout the day were lacking. Third, self-reported information may not accurately capture unintentional naps or forgotten napping instances, which led to more credible information not being available for our study. Future studies would benefit from including more reliable and objective measures. Furthermore, while we adjusted for several covariates in our models, there may still be some potential confounders, such as sleep quality, night shifts (which may lead to longer and more frequent daytime naps), work status and ApoE genotypes. A genome-wide association study revealed strong associations between two well-established approaches to BA estimation and ApoE (43). In addition, participants might alter their napping behavior at follow-up, which deserves to be followed in the future. Finally, we could not completely rule out reverse causality, a published study found a bidirectional relationship between daytime napping and Alzheimer’s dementia, and the complicated mechanisms involved need to be further investigated (44).

In summary, we discovered the association between napping, aging, and cognitive function among a nationally representative sample of middle-aged and older Chinese individuals. The significant mediation effect revealed a pathway from napping to cognitive decline through the acceleration of biological age. Our study underscores that daytime napping could work as an early sign or risk factor for biological aging and clinically significant cognitive impairment. The findings highlight that daytime napping behavior, as a lifestyle, is a cost-effective intervention that can be easily promoted to middle-aged and older populations. Future research with more objective measures of napping behaviors and more methods to quantify biological age is required to validate our findings.

## Data availability statement

The datasets presented in this study can be found in online repositories. The names of the repository/repositories and accession number(s) can be found at: http://charls.pku.edu.cn/.

## Ethics statement

The studies involving humans were approved by the Ethics Committee of Peking University. The studies were conducted in accordance with the local legislation and institutional requirements. The participants provided their written informed consent to participate in this study.

## Author contributions

HW: Conceptualization, Writing – original draft, Writing – review & editing. LH: Funding acquisition, Writing – review & editing. SZ: Writing – review & editing. YZ: Conceptualization, Supervision, Writing – original draft, Writing – review & editing. YL: Writing – review & editing.
