# Supplementary material for: Daytime napping, biological aging and cognitive function among middle-aged and older Chinese: insights from the China health and retirement longitudinal study
Source: Front Public Health. 2023 Nov 17;11:1294948. doi: 10.3389/fpubh.2023.1294948 (PMC10693455; doi:10.3389/fpubh.2023.1294948)
Supplement: Supplementary file 1 [file Data_Sheet_1.docx]

**Supplementary methods**

**Calculating biological age measures**

Klemera and Doubal biological age (KDM-BA)

The KDM algorithm is derived from a series of regressions of each individual’s biomarkers on chronological age (CA) in a reference population(1). The following equation was used to calculate KDM-BA,

| $KDM-BA=\frac{\sum_{j=1}^{m} \left( x_{j}-q_{j} \right)\frac{k_{j}}{s_{j}^{2}}+\frac{CA}{S_{BA}^{2}}}{\sum_{j=1}^{m} \left( \frac{k_{j}}{s_{j}} \right)^{2}+\frac{1}{S_{BA}^{2}}}$ | (1) |
| --- | --- |

Where CA stands for chronological age and $x_{j}$ is the biomarker j measured for an individual in the CHARLS. The biomarker list included creatinine, high-sensitivity C-reactive protein, total cholesterol, triglycerides, glycosylated hemoglobin, urea nitrogen, platelets, and systolic blood pressure. The parameters were trained using data from the China Nutrition and Health Survey (CHNS) cohort and then projected onto CHARLS. For each biomarker *j*, the parameters *k*, *q*, and *s* are estimated from a regression of chronological age on the biomarker in the CHNS. *k*, *q*, and *s* are the regression intercept, slope, and root mean squared error, respectively. *S*_BA_ is a scaling factor equal to the square root of the variance in chronological age explained by the biomarker set in the CHNS.

Physiological dysregulation (PD)

PD was derived from the Mahalanobis distance by extracting information on multiple biomarkers with respect to a reference or mean baseline population. In our study, we selected a sample aged 20–39 years from the CHNS as the reference population to parameterize the algorithms, the following equation was used to calculate PD,

| $\boldsymbol{D}_{\boldsymbol{M}}\left( \vec{\boldsymbol{x}} \right)\boldsymbol{=}\sqrt{\left( \vec{\boldsymbol{x}}\boldsymbol{-}\vec{\boldsymbol{u}} \right)^{\boldsymbol{T}}\boldsymbol{S}^{\boldsymbol{-1}}\left( \vec{\boldsymbol{x}}\boldsymbol{-}\vec{\boldsymbol{u}} \right)}$ | (2) |
| --- | --- |

Where, x is a multivariate observation (all the biomarkers for a given individual at a given time point) and *μ* is the equivalent length vector of reference sample means for each variable. *S* is the reference sample variance–covariance matrix for the variables.

**Reference**

1. Klemera P, Doubal S. A new approach to the concept and computation of biological age. Mech Ageing Dev. (2006) 127:240-8. doi: 10.1016/j.mad.2005.10.004.

**Supplementary Table 1. Follow-up information by characteristics at baseline.**

| Characteristics | Number of participants at baseline | Number of participants with at one remeasurement | Mean Follow-up (years) | Median follow-up (IQR) (years) |
| --- | --- | --- | --- | --- |
| All | 6647 | 6031 | 6.56 | 7.00[6.92-7.00] |
| Male | 3363 | 3099 | 6.50 | 7.00[6.92-7.00] |
| Female | 3284 | 2932 | 6.62 | 7.00[6.92-7.00] |
| Age (years) |  |  |  |  |
| <65 | 5085 | 4743 | 6.64 | 7.00[6.92-7.00] |
| ≥65 | 1562 | 1288 | 6.26 | 7.00[6.92-7.00] |
| Residence |  |  |  |  |
| Urban | 2489 | 2265 | 6.44 | 7.00[6.92-7.00] |
| Rural | 4158 | 3766 | 6.63 | 7.00[6.92-7.00] |
| Education level ^a^ |  |  |  |  |
| Low | 4327 | 3818 | 6.56 | 7.00[6.92-7.00] |
| Middle | 1681 | 1599 | 6.57 | 7.00[6.92-7.00] |
| High | 639 | 614 | 6.56 | 7.00[6.92-7.00] |
| Marital status |  |  |  |  |
| Married | 5675 | 5205 | 6.60 | 7.00[6.92-7.00] |
| Others | 972 | 826 | 6.30 | 7.00[6.92-7.00] |
| BMI (kg/m^2^) ^b^ |  |  |  |  |
| <18.5 | 378 | 311 | 6.51 | 7.00[6.92-7.00] |
| 18.5-23.9 | 3446 | 3127 | 6.53 | 7.00[6.92-7.00] |
| 24-28 | 2017 | 1849 | 6.61 | 7.00[6.92-7.00] |
| ≥28 | 806 | 744 | 6.59 | 7.00[6.92-7.00] |
| Smoking status |  |  |  |  |
| Current smokers | 2811 | 2568 | 6.50 | 7.00[6.92-7.00] |
| Nonsmokers | 3836 | 3463 | 6.60 | 7.00[6.92-7.00] |
| Drinking status |  |  |  |  |
| Non-drinker | 4321 | 3873 | 6.57 | 7.00[6.92-7.00] |
| Less than once a month | 543 | 511 | 6.56 | 7.00[6.92-7.00] |
| More than once a month | 1783 | 1647 | 6.54 | 7.00[6.92-7.00] |
| Night sleep duration |  |  |  |  |
| <7 h | 2802 | 2596 | 6.57 | 7.00[6.92-7.00] |
| 7-8h | 3335 | 2984 | 6.54 | 7.00[6.92-7.00] |
| >8h | 510 | 451 | 6.63 | 7.00[6.92-7.00] |
| Depressive symptoms ^c^ |  |  |  |  |
| Yes | 1740 | 1520 | 6.55 | 7.00[6.92-7.00] |
| No | 4907 | 4511 | 6.56 | 7.00[6.92-7.00] |
| Chronic diseases |  |  |  |  |
| Hypertension |  |  |  |  |
| Yes | 1657 | 1484 | 6.51 | 7.00[6.92-7.00] |
| No | 4990 | 4547 | 6.57 | 7.00[6.92-7.00] |
| Diabetes |  |  |  |  |
| Yes | 398 | 359 | 6.41 | 7.00[6.92-7.00] |
| No | 6249 | 5672 | 6.57 | 7.00[6.92-7.00] |
| Heart disease |  |  |  |  |
| Yes | 776 | 708 | 6.44 | 7.00[6.92-7.00] |
| No | 5871 | 5323 | 6.57 | 7.00[6.92-7.00] |
| Stroke  Yes  No | 129  6518 | 113  5918 | 6.45  6.56 | 7.00[6.92-7.00]  7.00[6.92-7.00] |
|  |  |  |  |  |

BMI, body mass index; ADL, activities of daily living; IADL, instrumental activities of daily living.

^a^ Low education level was defined as elementary school or below; middle was defined as school, high school, and vocational school; and high was defined as an associate degree or above.

^b^ BMI was calculated as weight in kilograms divided by height in meters squared.

^c^ Depressive symptoms were defined as a ten-item Center for Epidemiologic Studies Depression (CES-D 10) scale score of 12 or above.

**Supplementary Table 2. The original scores of cognitive domains during follow-up.**

| Cognitive domain | Baseline (N=6031) | Wave 2 (N=5069) | Wave 3 (N=5114) | Wave 4 (N=3773) |
| --- | --- | --- | --- | --- |
| Memory | 7.54 ± 3.26 | 7.77 ± 3.25 | 7.14 ± 3.43 | 8.05 ± 4.02 |
| Orientation | 3.19 ± 0.97 | 3.21 ± 1.01 | 3.15 ± 1.03 | 3.15 ± 0.93 |
| Executive | 5.94 ± 2.28 | 5.94 ± 2.24 | 5.83 ± 2.25 | 5.52 ± 2.26 |

Data are expressed as mean ± standard deviation (SD).

**Supplementary Table 3. Cross-sectional associations of napping duration with three cognitive domains.**

| Napping group | Memory z scores | | Orientation z scores | | Executive z scores | |
| --- | --- | --- | --- | --- | --- | --- |
|  | LSM ^a^ (95%CI) | *p* value | LSM^a^ (95% CI) | *p* value | LSN ^a^ (95% CI) | *p* value |
| Non-nappers  (0 minutes) | -0.041 (-0.108,0.025) | 0.328 | -0.070 (-0.135, -0.005) | 0.030 | -0.071 (-0.138, -0.003) | 0.036 |
| Short nappers  (≤ 30 minutes) | 0.060 (-0.022,0.143) | 0.211 | -0.012 (-0.093,0.068) | 0.947 | -0.013 (-0.096,0.071) | 0.946 |
| Moderate nappers  (30-90 minutes) | Reference | - | Reference | - | Reference | - |
| Extended nappers  (≥ 90 minutes) | -0.084 (-0.179,0.010) | 0.100 | -0.048 (-0.140, 0.045) | 0.471 | -0.102 (-0.197, -0.007) | 0.031 |

LSM, least-squares means; KDM-BAacc, Klemera and Doubal method-biological age acceleration; PD, physiological dysregulation; CI, confidence interval.

^a^ after adjusting for age, sex, education level, residence, BMI, marital status, current smoking and drinking status, night sleep duration, depressive symptoms, and self-reported chronic diseases.

**Supplementary Table 4. Longitudinal associations of baseline napping duration and biological age measures with memory function.**

| Variable | Model 1 | | Model 2 | | Model 3a | | Model 3b | |
| --- | --- | --- | --- | --- | --- | --- | --- | --- |
|  | β (95%CI) | *p* value | β (95%CI) | *p* value | β (95%CI) | *p* value | β (95%CI) | *p* value |
| Napping duration, min |  |  |  |  |  |  |  |  |
| 0 | -0.078 (-0.132, -0.025) | 0.004 | -0.025 (-0.081,0.022) | 0.268 | -0.022 (-0.079,0.025) | 0.388 | -0.023 (-0.079,0.023) | 0.289 |
| ≤ 30 | 0.031  (-0.035,0.098) | 0.356 | 0.010 (-0.054,0.074) | 0.752 | 0.010 (-0.054,0.074) | 0.766 | 0.010 (-0.053,0.074) | 0.750 |
| 30-90 | Reference | - | Reference | - | Reference | - | Reference | - |
| ≥ 90 | -0.087 (-0.164, -0.011) | 0.025 | -0.087 (-0.160, -0.014) | 0.019 | -0.087 (-0.160, -0.014) | 0.020 | -0.087 (-0.161, -0.014) | 0.019 |
| Time since baseline (years) | -0.028 (-0.038, -0.018) | <0.001 | -0.027 ( -0.037, -0.018) | <0.001 | -0.027 (-0.037, -0.018) | <0.001 | -0.027 (-0.038, -0.019) | <0.001 |
| Napping duration x Time since baseline |  |  |  |  |  |  |  |  |
| 0 | -0.009 (-0.021, 0.003) | 0.126 | -0.009 (-0.022, 0.003) | 0.107 | -0.009 (-0.022,0.002) | 0.103 | -0.009 (-0.021, 0.002) | 0.128 |
| ≤ 30 | 0.004  (-0.011,0.020) | 0.561 | 0.004 (-0.011,0.019) | 0.601 | 0.004 (-0.011,0.019) | 0.642 | 0.005 (-0.010,0.020) | 0.551 |
| 30-90 | Reference | - | Reference | - | Reference | - | Reference | - |
| ≥ 90 | -0.009 (-0.026,0.008) | 0.314 | -0.009 (-0.026,0.008) | 0.297 | -0.009 (-0.026, 0.008) | 0.310 | -0.008 (-0.025,0.009) | 0.358 |
| KDM-BAacc | - | - | - | - | -0.003 (-0.011,0.004) | 0.400 | - | - |
| KDM-BAacc x Time since baseline | - | - | - | - | -0.002 (-0.004, -0.000) | 0.016 | - | - |
| PD | - | - | - | - | - | - | -0.004 (-0.025,0.018) | 0.746 |
| PD x Time since baseline | - | - | - | - | - | - | -0.011 (-0.016, -0.006) | <0.001 |

KDM-BAacc, Klemera and Doubal method-biological age acceleration; PD, physiological dysregulation; CI, confidence interval. Model 1 was adjusted for age and sex. Model 2 was additionally adjusted for education level, residence, BMI, marital status, current smoking and drinking status, night sleep duration, depressive symptoms, and self-reported chronic diseases based on Model 1. Model 3a was additionally adjusted for KDM-BAacc, based on Model 2. Model 3b was additionally adjusted for PD, based on Model 2.

**Supplementary Table 5. Longitudinal associations of baseline napping duration and biological age measures with orientation function.**

| Variable | Model 1 | | Model 2 | | Model 3a | | Model 3b | |
| --- | --- | --- | --- | --- | --- | --- | --- | --- |
|  | β (95%CI) | *p* value | β (95%CI) | *p* value | β (95%CI) | *p* value | β (95%CI) | *p* value |
| Napping duration, min |  |  |  |  |  |  |  |  |
| 0 | -0.108 (-0.163, -0.054) | <0.001 | -0.061 (-0.112, -0.009) | 0.021 | -0.060 (-0.111, -0.008) | 0.023 | -0.057 (-0.109, -0.006) | 0.028 |
| ≤ 30 | 0.016  (-0.056,0.080) | 0.731 | -0.016 (-0.080,0.051) | 0.618 | -0.016 (-0.078,0.050) | 0.620 | -0.013 (-0.077,0.051) | 0.686 |
| 30-90 | Reference | - | Reference | - | Reference | - | Reference | - |
| ≥ 90 | -0.106 (-0.184, -0.028) | 0.008 | -0.104 (-0.177, -0.031) | 0.005 | -0.103 (-0.176, -0.031) | 0.005 | -0.102 (-0.175, -0.029) | 0.006 |
| Time since baseline (years) | -0.041 (-0.050, -0.032) | <0.001 | -0.040 (-0.049, -0.032) | <0.001 | -0.040 (-0.049, -0.032) | <0.001 | -0.041 (-0.049, -0.032) | <0.001 |
| Napping duration x Time since baseline |  |  |  |  |  |  |  |  |
| 0 | 0.001 (-0.010,0.012) | 0.792 | 0.001 (-0.010,0.012) | 0.848 | 0.001 ( -0.010,0.011) | 0.855 | 0.001 (-0.010,0.012) | 0.825 |
| ≤ 30 | -0.004  (-0.017,0.019) | 0.570 | -0.004 (-0.018,0.009) | 0.555 | -0.004 (-0.018,0.010) | 0.539 | -0.004 (-0.017,0.010) | 0.575 |
| 30-90 | Reference | - | Reference | - | Reference | - | Reference | - |
| ≥ 90 | 0.002 (-0.017,0.014) | 0.837 | -0.002 (-0.017,0.013) | 0.792 | -0.002 (-0.018,0.013) | 0.784 | -0.002 (-0.018,0.014) | 0.803 |
| KDM-BAacc | - | - | - | - | -0.001 (-0.008,0.007) | 0.857 | - | - |
| KDM-BAacc x Time since baseline | - | - | - | - | -0.001 (-0.002,0.001) | 0.288 | - | - |
| PD | - | - | - | - | - | - | -0.031 (-0.053, -0.010) | 0.004 |
| PD x Time since baseline | - | - | - | - | - | - | -0.002 (-0.007,0.002) | 0.288 |

KDM-BAacc, Klemera and Doubal method-biological age acceleration; PD, physiological dysregulation; CI, confidence interval. Model 1 was adjusted for age and sex. Model 2 was additionally adjusted for education level, residence, BMI, marital status, current smoking and drinking status, night sleep duration, depressive symptoms, and self-reported chronic diseases based on Model 1. Model 3a was additionally adjusted for KDM-BAacc, based on Model 2. Model 3b was additionally adjusted for PD, based on Model 2.

**Supplementary Table 6. Longitudinal associations of baseline napping duration and biological age measures with executive function.**

| Variable | Model 1 | | Model 2 | | Model 3a | | Model 3b | |
| --- | --- | --- | --- | --- | --- | --- | --- | --- |
|  | β (95%CI) | *p* value | β (95%CI) | *p* value | β (95%CI) | *p* value | β (95%CI) | *p* value |
| Napping duration, min |  |  |  |  |  |  |  |  |
| 0 | -0.127 (-0.179, -0.074) | <0.001 | -0.080 (-0.131, -0.032) | 0.001 | -0.077 (-0.127, -0.029) | 0.002 | -0.077 ( -0.130, -0.029) | 0.002 |
| ≤ 30 | 0.005  (-0.060,0.070) | 0.877 | -0.019 (-0.079,0.044) | 0.524 | -0.018 (-0.081,0.042) | 0.533 | -0.019 (-0.077,0.045) | 0.545 |
| 30-90 | Reference | - | Reference | - | Reference | - | Reference | - |
| ≥ 90 | -0.062 (-0.137,0.013) | 0.106 | -0.060 (-0.130,0.011) | 0.096 | -0.059 (-0.130,0.011) | 0.099 | -0.058 (-0.128,0.013) | 0.108 |
| Time since baseline (years) | -0.058 (-0.066, -0.050) | <0.001 | -0.058 (-0.066, -0.050) | <0.001 | -0.058 (-0.066, -0.050) | <0.001 | -0.059 (-0.067, -0.051) | <0.001 |
| Napping duration x Time since baseline |  |  |  |  |  |  |  |  |
| 0 | -0.005 (-0.015,0.005) | 0.353 | -0.005 (-0.015,0.005) | 0.302 | -0.005 (-0.016,0.005) | 0.296 | -0.005 (-0.015,0.005) | 0.330 |
| ≤ 30 | -0.003  (-0.016,0.010) | 0.634 | -0.003 (-0.016,0.009) | 0.595 | -0.003 (-0.013,0.010) | 0.600 | -0.003 (-0.016,0.010) | 0.625 |
| 30-90 | Reference | - | Reference | - | Reference | - | Reference | - |
| ≥ 90 | -0.008 (-0.023,0.006) | 0.263 | -0.009 (-0.024,0.006) | 0.235 | -0.009 (-0.023,0.006) | 0.245 | -0.008 (-0.023,0.006) | 0.270 |
| KDM-BAacc | - | - | - | - | -0.009 (-0.017, -0.002) | 0.012 | - | - |
| KDM-BAacc x Time since baseline | - | - | - | - | -0.001 (-0.002,0.001) | 0.231 | - | - |
| PD | - | - | - | - | - | - | -0.033 (-0.054, -0.012) | 0.002 |
| PD x Time since baseline | - | - | - | - | - | - | -0.005 (-0.009, -0.000) | 0.040 |

KDM-BAacc, Klemera and Doubal method-biological age acceleration; PD, physiological dysregulation; CI, confidence interval. Model 1 was adjusted for age and sex. Model 2 was additionally adjusted for education level, residence, BMI, marital status, current smoking and drinking status, night sleep duration, depressive symptoms, and self-reported chronic diseases based on Model 1. Model 3a was additionally adjusted for KDM-BAacc, based on Model 2. Model 3b was additionally adjusted for PD, based on Model 2.

**Supplementary Table 7. Cross-sectional associations of napping with global cognitive function or biological age measures.**

| Napping group | Global z scores | | KDM-BAacc | | PD | |  |
| --- | --- | --- | --- | --- | --- | --- | --- |
|  | LS means ^a^ (95%CI) | *p* value | LS means ^a^ (95%CI) | *p* value | LS means ^a^ (95%CI) | *p* value |  |
|  |  |  |  |  |  |  |  |
| Non-nappers | Reference | - | Reference | - | Reference | - |  |
| Nappers | 0.048 (0.018, 0.079) | 0.002 | -0.300 (-0.434,0.166) | <0.001 | -0.060 (-0.108, -0.013) | 0.012 |  |

KDM-BAacc, Klemera and Doubal method-biological age acceleration; PD, physiological dysregulation; CI, confidence interval.

^a^ after adjusting for age, sex, education level, residence, BMI, marital status, current smoking and drinking status, night sleep duration, depressive symptoms, and self-reported chronic diseases.

**Supplementary Table 8. Longitudinal associations of baseline napping and biological age measures with global cognitive decline.**

| Variable | Model 1 | | Model 2 | | Model 3a | | Model 3b | |
| --- | --- | --- | --- | --- | --- | --- | --- | --- |
|  | β(95%CI) | *p*-value | β(95% CI) | *p*-value | β(95% CI) | *p*-value | β(95% CI) | *p*-value |
| Napping groups |  |  |  |  |  |  |  |  |
| Non-nappers | Reference | - | Reference | - | Reference | - | Reference | - |
| Nappers | 0.095 (0.059,0.132) | <0.001 | 0.034 (0.001,0.067) | 0.047 | 0.029 (-0.003,0.068) | 0.091 | 0.029 (-0.002,0.065) | 0.094 |
| Time since baseline, years | -0.074 ( -0.080, -0.067) | <0.001 | -0.071 (-0.077, -0.064) | <0.001 | -0.071 (-0.079, -0.065) | <0.001 | -0.072 (-0.079, -0.066) | <0.001 |
| Napping duration x Time since baseline |  |  |  |  |  |  |  |  |
| Non-nappers | Reference | - | Reference | - | Reference | - | Reference | - |
| Nappers | 0.010 (0.001,0.018) | 0.028 | 0.009 (0.001,0.017) | 0.035 | 0.009 (0.0001,0.019) | 0.034 | -0.012 (0.001, 0.017) | <0.001 |
| KDM-BAacc | - | - | - | - | -0.007 (-0.013, -0.001) | 0.021 | - | - |
| KDM-BAacc x Time since baseline | - | - | - | - | -0.001 (-0.002,0.000) | 0.065 | - | - |
| PD | - | - | - | - | - | - | -0.027 (-0.045, -0.010) | 0.002 |
| PD x Time since baseline | - | - | - | - | - | - | -0.012 ( -0.016, -0.007) | <0.001 |

KDM-BAacc, Klemera and Doubal method-biological age acceleration; PD, physiological dysregulation; CI, confidence interval. Model 1 was adjusted for age and sex. Model 2 was additionally adjusted for education level, residence, BMI, marital status, current smoking and drinking status, night sleep duration, depressive symptoms, and self-reported chronic diseases based on Model 1. Model 3a was additionally adjusted for KDM-BAacc, based on Model 2. Model 3b was additionally adjusted for PD, based on Model 2.

**Supplementary Table 9. Longitudinal associations of baseline napping duration and biological age measures with global cognitive decline (adjusted for family clustering effect).**

|  | Model 1 | | Model 2 | | Model 3a | | Model 3b | |
| --- | --- | --- | --- | --- | --- | --- | --- | --- |
|  | β (95%CI) | *p* value | β (95%CI) | *p* value | β (95%CI) | *p* value | β (95%CI) | *p* value |
| Napping duration, min |  |  |  |  |  |  |  |  |
| 0 | -0.096 (-0.142, -0.051) | <0.001 | -0.050 (-0.091, -0.008) | 0.020 | -0.039 (-0.093, -0.012) | 0.021 | -0.047 (-0.088, -0.005) | 0.028 |
| ≤ 30 | 0.009  (-0.048,0.066) | 0.759 | -0.015 (-0.066,0.049) | 0.571 | -0.025 (-0.060,0.040) | 0.337 | -0.014 (-0.066,0.037) | 0.586 |
| 30-90 | Reference | - | Reference | - | Reference | - | Reference | - |
| ≥ 90 | -0.084 (-0.149, -0.019) | 0.011 | -0.079 (-0.138, -0.020) | 0.009 | -0.073 (-0.146, -0.026) | 0.014 | -0.078 (-0.136, -0.019) | 0.010 |
| Time since baseline (years) | -0.060 (-0.068, -0.051) | <0.001 | -0.058 (-0.066, -0.050) | <0.001 | -0.057 (-0.066, -0.050) | <0.001 | -0.059 (-0.068, -0.051) | <0.001 |
| Napping duration x Time since baseline |  |  |  |  |  |  |  |  |
| 0 | -0.014 (-0.024, -0.003) | 0.011 | -0.013 (-0.024, -0.003) | 0.014 | -0.012 (-0.024, -0.003) | 0.012 | -0.013 (-0.023, -0.002) | 0.017 |
| ≤ 30 | -0.001 (-0.014, 0.013) | 0.957 | 0.000 (-0.013, 0.013) | 0.989 | -0.000 (-0.013, 0.013) | 0.989 | 0.000 (-0.012, 0.014) | 0.920 |
| 30-90 | Reference | - | Reference | - | Reference | - | Reference | - |
| ≥ 90 | -0.021 (-0.036, -0.006) | 0.007 | -0.020 (0.035, -0.005) | 0.008 | -0.020 (-0.035, -0.005) | 0.008 | -0.019 (-0.034, -0.004) | 0.012 |
| KDM-BAacc | - | - | - | - | -0.005 (-0.015, -0.001) | 0.016 | - | - |
| KDM-BAacc x Time since baseline | - | - | - | - | -0.001 (-0.008,0.003) | 0.121 | - | - |
| PD | - | - | - | - | - | - | -0.024 (-0.041, -0.006) | 0.007 |
| PD x Time since baseline | - | - | - | - | - | - | -0.011 (-0.016, -0.007) | <0.001 |

KDM-BAacc, Klemera and Doubal method-biological age acceleration; PD, physiological dysregulation; CI, confidence interval. Model 1 was adjusted for age and sex. Model 2 was additionally adjusted for education level, residence, BMI, marital status, current smoking and drinking status, night sleep duration, depressive symptoms, and self-reported chronic diseases based on Model 1. Model 3a was additionally adjusted for KDM-BAacc, based on Model 2. Model 3b was additionally adjusted for PD, based on Model 2.


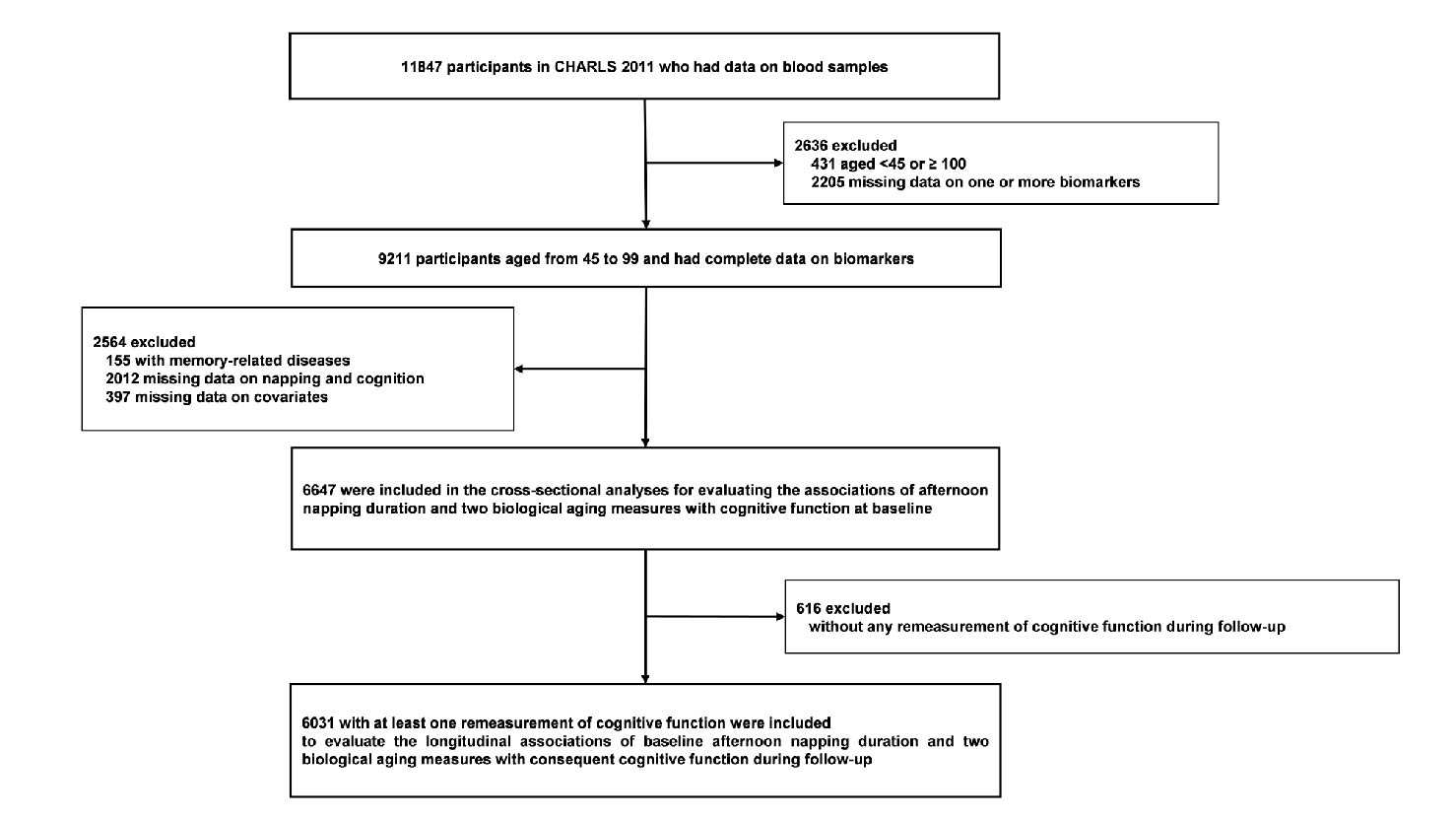


**Supplementary Figure 1. The flowchart of the participant selection in the study.**


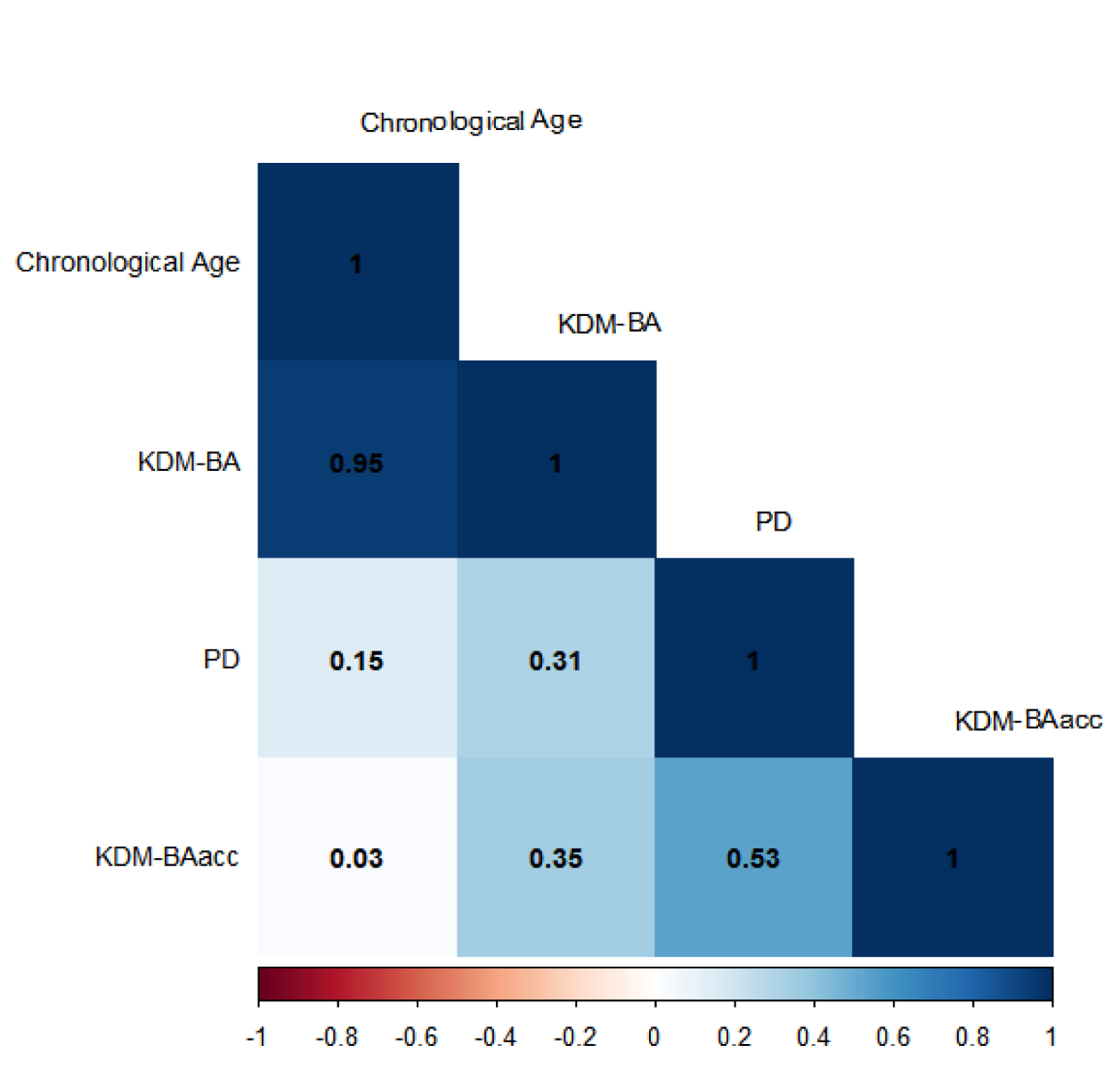


**Supplementary Figure 2. Correlation matrix of chronological age and biological age measures.**

KDM_BA, Klemera and Doubal method-biological age; KDM-BAacc, Klemera and Doubal method-biological age acceleration; PD, physiological dysregulation.


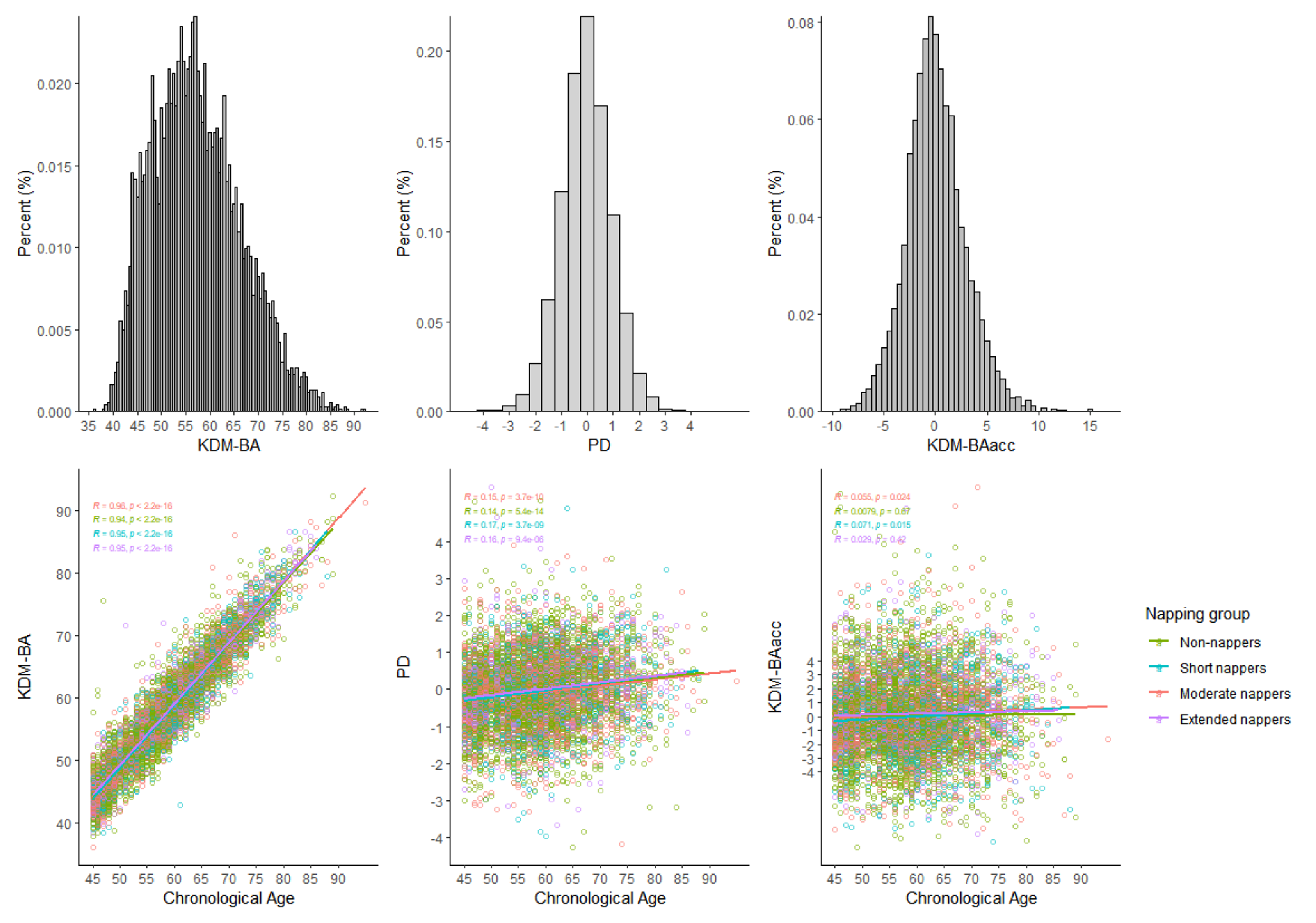


**Supplementary Figure 3.** **Distribution of biological age measures, and correlation of chronological age with biological age measures in each napping group at baseline.**

KDM_BA, Klemera and Doubal method-biological age; KDM-BAacc, Klemera and Doubal method-biological age acceleration; PD, physiological dysregulation.


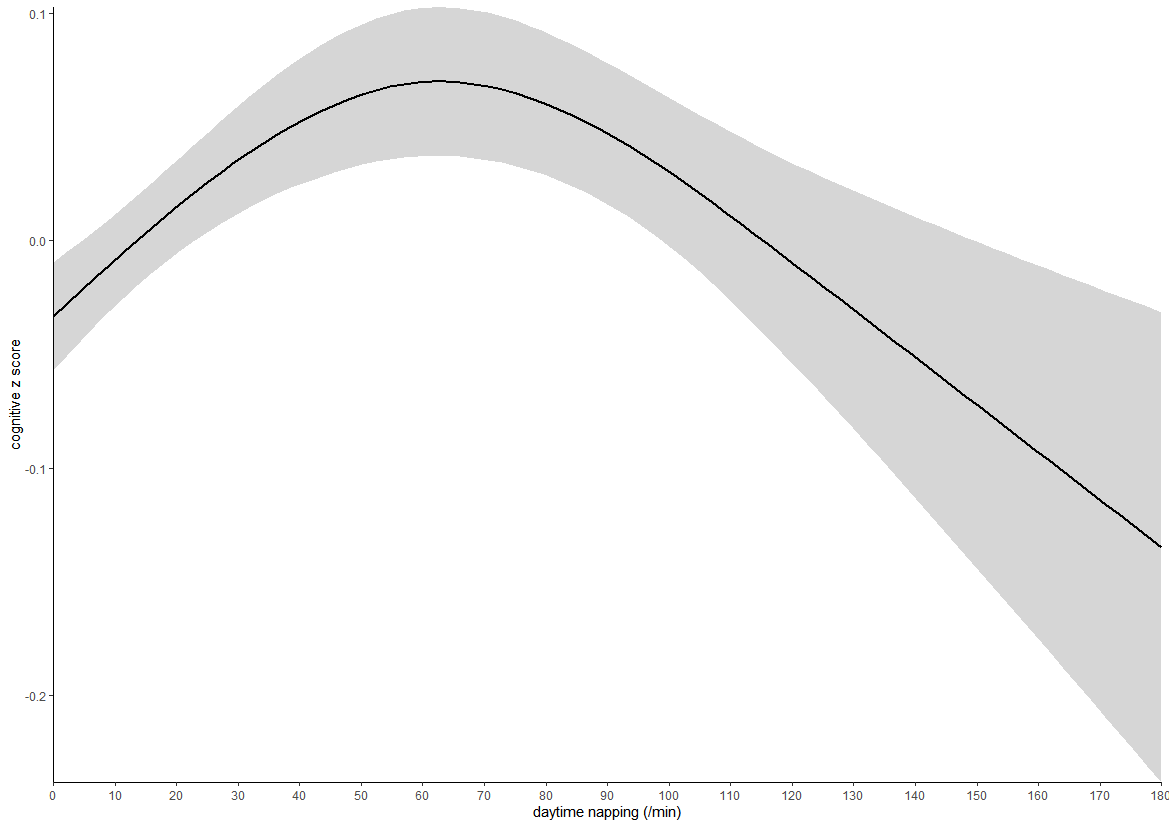


**Supplementary Figure 4. The non-linear association between daytime napping and cognitive z scores.**
